# Impact of age and comorbidities on the efficacy and tolerability of bosutinib in previously treated patients with chronic myeloid leukemia: results from the phase 4 BYOND study

**DOI:** 10.1038/s41375-023-02080-y

**Published:** 2023-11-25

**Authors:** Gianantonio Rosti, Tim H. Brümmendorf, Björn T. Gjertsen, Pilar Giraldo-Castellano, Fausto Castagnetti, Carlo Gambacorti-Passerini, Thomas Ernst, Huadong Zhao, Luke Kuttschreuter, Simon Purcell, Francis J. Giles, Andreas Hochhaus

**Affiliations:** 1 IRCCS Istituto Romagnolo per lo Studio dei Tumori (IRST) “Dino Amadori”, Meldola, Italy; 2 Universitätsklinikum RWTH Aachen, Aachen, Germany; 3 Center for Integrated Oncology Aachen Bonn Cologne Düsseldorf (CIO ABCD), Aachen, Germany; 4 Haukeland University Hospital, Department of Medicine, Hematology Section, Helse Bergen, and Centre for Cancer Biomarkers CCBIO, Department of Clinical Science, University of Bergen, Bergen, Norway; 5 CIBER Enfermedades Raras (CIBERER), Miguel Servet University Hospital, Zaragoza, Spain; 6 Institute of Hematology “Seràgnoli”, IRCCS Azienda Ospedaliero-Universitaria di Bologna, Bologna, Italy; 7 Department of Medical and Surgical Sciences, University of Bologna, Bologna, Italy; 8 University of Milano-Bicocca, Monza, Italy; 9 Klinik für Innere Medizin II, Universitätsklinikum Jena, Jena, Germany; 10 Pfizer (China) R&D Co., Ltd., Shanghai, China; 11 Pfizer Ltd, Oxford, UK; 12 Pfizer Ltd, London, UK; 13 Developmental Therapeutics Consortium, Chicago, IL USA

**Keywords:** Chronic myeloid leukaemia, Phase IV trials

## Abstract

In the phase 4 BYOND trial, patients with pretreated chronic myeloid leukemia (CML) received bosutinib (starting dose: 500 mg/day). Efficacy and safety after ≥3 years of follow-up in 156 patients with Philadelphia chromosome–positive chronic phase CML by age and Charlson Comorbidity Index scores (without the age component; mCCI) is reported. Cumulative major molecular response rates at any time on treatment were 73.6%, 64.5%, and 74.1% in patients <65, 65–74, and ≥75 years of age, and 77.9%, 63.0%, and 59.3% in patients with mCCI scores 2, 3, and ≥4, respectively. Patients <65, 65–74, and ≥75 years of age experienced grade 3/4 treatment-emergent adverse events (TEAEs) at rates of 74.7%, 78.8%, and 96.4% and permanent discontinuations due to AEs at rates of 22.1%, 39.4%, and 46.4%, respectively. In patients with mCCI 2, 3, and ≥4, respective rates of grade 3/4 TEAEs were 77.8%, 77.8%, and 86.7%, and permanent discontinuations due to AEs were 25.3%, 33.3%, and 43.3%. In conclusion, a substantial proportion of patients maintained/achieved cytogenetic and molecular responses across age groups and mCCI scores. Older patients (≥75 years) and those with high comorbidity burden (mCCI ≥4) may require more careful monitoring due to the increased risk of TEAEs. Clinicaltrials.gov: NCT02228382.

## Introduction

The development of *BCR*::*ABL1*-targeting tyrosine kinase inhibitors (TKIs) has led to substantial improvements in clinical outcomes for patients with chronic myeloid leukemia (CML) [1–6]. Bosutinib is a second-generation TKI approved for the treatment of adult patients with newly diagnosed Philadelphia chromosome–positive (Ph+) chronic phase (CP) CML, or for patients with Ph+ CP, accelerated phase (AP), or blast phase CML with resistance or intolerance to prior TKI therapy [7, 8]. Approval of bosutinib for patients with Ph+ CP CML previously treated with at least one TKI was based on results from a phase 1/2 study [9, 10]. With ≥5 years of follow-up, bosutinib 500 mg once daily (QD) demonstrated durable efficacy and manageable toxicity in this patient population [11]. In addition, patients receiving second- or third-line bosutinib have been shown to largely maintain health-related quality of life with long-term treatment [12].

The phase 4 BYOND study was designed to provide further information on the efficacy and safety of bosutinib in patients with CP CML who were resistant/intolerant to prior TKIs [13]. At study completion (median follow-up, 47.8 months), 48.1% of patients with Ph+ CP CML remained on treatment, and 68.6% completed the study. Among evaluable patients, 81.1%, 71.8%, and 59.7% attained or maintained complete cytogenetic response (CCyR), major molecular response (MMR), and deep molecular response (MR^4^), respectively, at any time on treatment. Long-term adverse events (AEs) were consistent with the known safety profile of bosutinib, and no new safety issues were identified [14].

In patients with CML, previous studies have reported an association between higher Charlson Comorbidity Index (CCI) score and reduced overall survival (OS) [15–20]. Certain comorbidities have also been associated with the development of specific toxicities during TKI treatment [21–27]. Prior to the advent of TKIs, older age was considered a negative prognostic factor for the treatment of CML [28]. While data are limited, a number of studies suggest that comorbidities, rather than age, influence survival outcomes in older patients with CML [28–31]. Therefore, this post hoc analysis of the BYOND study was performed to determine the impact of age and comorbidities on the efficacy and tolerability of bosutinib in previously treated patients with Ph+ CP CML.

## Methods

### Study design and patients

BYOND (ClinicalTrials.gov, NCT02228382) was an open-label, nonrandomized, single-arm phase 4 study of bosutinib for which the methods have been previously published [13]. Briefly, patients were aged ≥18 years and had a cytogenetic or PCR-based diagnosis of Ph+ CML, or *BCR*::*ABL1* positive if Ph– (from initial diagnosis), and prior treatment with at least one TKI. Any CML phase was permitted as long as the patient was resistant/intolerant to prior TKIs [13]. Patients received bosutinib 500 mg once daily (QD) as a starting dose. Dose escalations and reductions were permitted. Patients received bosutinib for up to 4 years, or until disease progression, unacceptable toxicity, consent withdrawal, death, or study discontinuation [13].

The study was conducted in accordance with the Declaration of Helsinki. All patients provided written informed consent before study procedures began, and the protocol was approved by institutional review boards at each study site.

### Efficacy and safety assessments

The primary endpoints of the BYOND study have been previously reported [13]. A post hoc subgroup analysis was performed in patients with Ph+ CP CML categorized by (i) age (<65, 65–74, and ≥75 years) and (ii) comorbidities. CCI scores (without the age component; mCCI) were derived from baseline data and patients grouped by mCCI scores 2 (CML only), 3, and ≥4 (Supplementary Methods) [32]. The following endpoints were assessed in the subgroups of age and mCCI score: cumulative CCyR, MMR (*BCR*::*ABL1* international scale [IS] ≤0.1%), MR^4^ (*BCR*::*ABL1* IS ≤ 0.01%), and MR^4.5^ (*BCR*::*ABL1* IS ≤ 0.0032%) at any time; on-treatment transformation to AP or blast phase CML; OS; safety; and patient-reported outcomes (PROs). Efficacy was assessed in patients who underwent dose reductions to 400, 300, or 200 mg/day (without further reduction) due to AEs.

Efficacy was evaluated by standard criteria [13]. Briefly, all bosutinib-treated patients with Ph+ CML with a valid baseline efficacy assessment for the respective endpoint (evaluable population) were included in the cytogenetic and molecular efficacy analyses. Analyses of cytogenetic response were based on the data from local laboratory assessment, whereas MR was assessed at an independent central laboratory. CCyR was imputed from MMR on a specific date if there was no valid cytogenetic assessment.

Treatment-emergent AEs (TEAEs), serious AEs, and laboratory evaluations were assessed up to 28 days after last dose. Events were graded according to the National Cancer Institute Common Terminology Criteria for Adverse Events v.4.0. The frequency and characteristics of AEs of special interest were analyzed by selecting Medical Dictionary for Regulatory Activities (MedDRA) system organ class high-level group, high-level and preferred terms, and standardized MedDRA queries to generate TEAE clusters (Supplementary Methods).

PROs were assessed using the Functional Assessment of Cancer Therapy-Leukemia (FACT-Leu) quality of life questionnaire at baseline, every 3 months for the first year, and every 6 months during years 2, 3, and 4 of treatment [33]. Higher scores represent better quality of life. The minimal important difference (MID) was defined as the smallest change in a PRO measure that was perceived by patients as beneficial or would result in a clinician considering change in treatment; MID was defined as 4–7 on the leukemia-specific subscale [34].

### Statistical analysis

Time-to-event endpoints (excluding OS) were estimated using cumulative incidence, adjusting for the competing risk of treatment discontinuation without the event. OS was estimated using the Kaplan–Meier method. Two-sided 95% confidence interval (CI) for response rate was determined using the exact binomial method. For Kaplan–Meier yearly probability estimates, two-sided 95% CI was based on Greenwood’s formula using a log(-log) transformation. All other data were summarized descriptively unless otherwise stated. All patients who received at least one dose of study drug (full analysis set) were included in the safety and PRO analyses.

This analysis was based on the final database lock, ≥3 years after the last enrolled patient; 90% of patients with Ph+ CP CML had a follow-up of 4 years or had already permanently discontinued.

## Results

### Patients and treatment

Of 163 patients who received bosutinib, 156 had Ph+ CP CML and were included in this analysis. Of those, 95 patients were <65 years of age, 33 were 65–74 years, and 28 were ≥75 years; and 99, 27, and 30 patients had mCCI scores of 2, 3, and ≥4, respectively. In the <65, 65–74, and ≥75 year age groups, respectively, the proportion of patients resistant to at least one prior TKI was 51.6%, 63.6%, and 42.9%, and the proportion of patients intolerant to all prior TKIs was 48.4%, 36.4%, and 57.1%. In patients with mCCI scores of 2, 3, and ≥4, respectively, the proportion of patients resistant to at least one prior TKI was 51.5%, 59.3%, and 50.0%, and the proportion of patients intolerant to all prior TKIs was 48.5%, 40.7%, and 50.0% (Table 1).

**Table 1:** Demographics and baseline characteristics in patients with Ph+ CP CML by age and comorbidities.

	By age			By comorbidities		
*n* (%)	<65 years *n* = 95	65–74 years *n* = 33	≥75 years *n* = 28	mCCI 2 *n* = 99	mCCI 3 *n* = 27	mCCI ≥ 4 *n* = 30
Male	47 (49.5)	21 (63.6)	13 (46.4)	45 (45.5)	15 (55.6)	21 (70.0)
Age, median (range), years	51.0 (20.0–64.0)	69.0 (65.0–74.0)	78.0 (75.0–89.0)	53.0 (20.0–89.0)	67.0 (38.0–87.0)	71.0 (54.0–85.0)
ECOG PS						
0	70 (73.7)	23 (69.7)	13 (46.4)	73 (73.7)	15 (55.6)	18 (60.0)
1	22 (23.2)	9 (27.3)	14 (50.0)	23 (23.2)	11 (40.7)	11 (36.7)
2	3 (3.2)	1 (3.0)	1 (3.6)	3 (3.0)	1 (3.7)	1 (3.3)
Number of prior TKIs						
1	32 (33.7)	7 (21.2)	5 (17.9)	33 (33.3)	6 (22.2)	5 (16.7)
2	31 (32.6)	15 (45.5)	14 (50.0)	37 (37.4)	11 (40.7)	12 (40.0)
3	27 (28.4)	10 (30.3)	9 (32.1)	25 (25.3)	10 (37.0)	11 (36.7)
4	5 (5.3)	1 (3.0)	0	4 (4.0)	0	2 (6.7)
Prior interferon alpha	8 (8.4)	2 (6.1)	1 (3.6)	6 (6.1)	3 (11.1)	2 (6.7)
Prior imatinib	81 (85.3)	32 (97.0)	28 (100.0)	87 (87.9)	25 (92.6)	29 (96.7)
Prior dasatinib	55 (57.9)	21 (63.6)	19 (67.9)	53 (53.5)	21 (77.8)	21 (70.0)
Prior nilotinib	51 (53.7)	16 (48.5)	12 (42.9)	52 (52.5)	9 (33.3)	18 (60.0)
Resistant to any prior TKI	49 (51.6)	21 (63.6)	12 (42.9)	51 (51.5)	16 (59.3)	15 (50.0)
Intolerant to all prior TKIs	46 (48.4)	12 (36.4)	16 (57.1)	48 (48.5)	11 (40.7)	15 (50.0)

*CML* chronic myeloid leukemia, *CP* chronic phase, *ECOG PS* Eastern Cooperative Oncology Group performance score, *mCCI* Charlson Comorbidity Index without age component, *Ph* Philadelphia chromosome, *TKI* tyrosine kinase inhibitor.

**Fig. 1:**
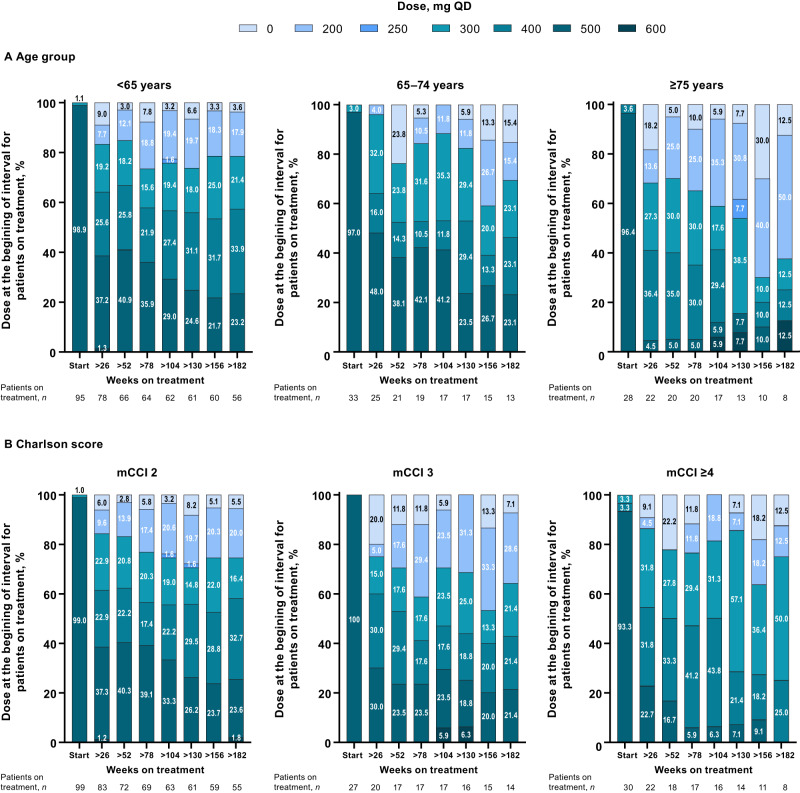
Bosutinib dose over time in patients with Ph+ CP CML. Patients grouped by (**A**) age and (**B**) comorbidities. CML chronic myeloid leukemia, CP chronic phase, mCCI Charlson Comorbidity Index without age component, Ph Philadelphia chromosome, QD once daily.

The median duration of treatment was 47.6, 34.7, and 27.1 months in patients <65, 65–74, and ≥75 years of age, respectively; corresponding median dose intensity was 320.9, 319.3, and 264.7 mg/day. Median treatment duration for patients with mCCI 2, 3, and ≥4 was 46.7, 44.9, and 24.8 months, respectively; corresponding median dose intensity was 320.8, 296.1, and 300.3 mg/day (Supplementary Table 1). Dose levels over time are presented in Fig. 1.

**Fig. 2:**
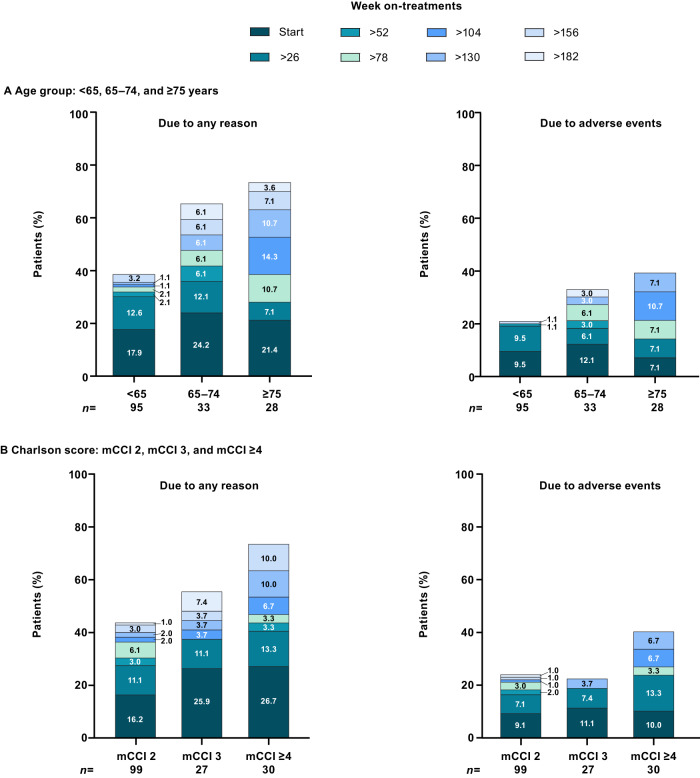
Reasons for permanent treatment discontinuation over time in patients with Ph+ CP CML. Patients grouped by (**A**) age and (**B**) comorbidities. CML chronic myeloid leukemia, CP chronic phase, mCCI Charlson Comorbidity Index without age component, Ph Philadelphia chromosome.

Bosutinib was permanently discontinued by 40.0%, 66.7%, and 75.0% of patients <65, 65–74, and ≥75 years of age, and 44.4%, 55.6%, and 73.3% of patients with mCCI 2, 3, and ≥4, respectively (Supplementary Table 1). The majority of discontinuations occurred in year 1 in patients <65 and 65–74 years of age and across CCI scores, and after year 1 in patients aged ≥75 years (Fig. 2 and Supplementary Table 2).

### Efficacy

In all patients evaluable for cytogenetic response, cumulative CCyR rates at any time on treatment were, respectively, 85.6%, 66.7%, and 82.6% in patients <65, 65–74, and ≥75 years of age and 87.8%, 72.0%, and 67.9% in patients with mCCI scores 2, 3, and ≥4 (Table 2). Among patients without baseline CCyR, cumulative CCyR rates at any time on treatment were, respectively, 75.0%, 35.7%, and 66.7% in patients <65, 65–74, and ≥75 years of age and 75.0%, 50.0%, and 40.0% in patients with mCCI scores 2, 3, and ≥4.

**Table 2:** Cumulative response rates at any time on treatment in patients with Ph+ CP CML by age and comorbidities (overall and excluding patients with the respective baseline response).

	By age			By comorbidities		
% (95% CI)	<65 years	65–74 years	≥75 years	mCCI 2	mCCI 3	mCCI ≥ 4
Cytogenetic response						
All evaluable patients, *n*	90	30	23	90	25	28
CCyR	85.6 (76.6–92.1)	66.7 (47.2–82.7)	82.6 (61.2–95.0)	87.8 (79.2–93.7)	72.0 (50.6–87.9)	67.9 (47.6–84.1)
Evaluable patients without baseline CCyR, *n*	32	14	6	32	10	10
CCyR	75.0 (56.6–88.5)	35.7 (12.8–64.9)	66.7 (22.3–95.7)	75.0 (56.6–88.5)	50.0 (18.7–81.3)	40.0 (12.2–73.8)
Molecular response						
All evaluable patients, *n*	91	31	27	95	27	27
MMR	73.6 (63.3–82.3)	64.5 (45.4–80.8)	74.1 (53.7–88.9)	77.9 (68.2–85.8)	63.0 (42.4–80.6)	59.3 (38.8–77.6)
MR^4^	59.3 (48.5–69.5)	51.6 (33.1–69.8)	66.7 (46.0–83.5)	62.1 (51.6–71.9)	55.6 (35.3–74.5)	51.9 (31.9–71.3)
MR^4.5^	47.3 (36.7–58.0)	48.4 (30.2–66.9)	51.9 (31.9–71.3)	48.4 (38.0–58.9)	48.1 (28.7–68.1)	48.1 (28.7–68.1)
Evaluable patients without baseline MMR, *n*	45	21	13	51	13	15
MMR	66.7 (51.0–80.0)	47.6 (25.7–70.2)	53.8 (25.1–80.8)	66.7 (52.1–79.2)	46.2 (19.2–74.9)	46.7 (21.3–73.4)
Evaluable patients without baseline MR^4^, *n*	65	26	21	70	22	20
MR^4^	52.3 (39.5–64.9)	42.3 (23.4–63.1)	61.9 (38.4–81.9)	52.9 (40.6–64.9)	54.5 (32.2–75.6)	45.0 (23.1–68.5)
Evaluable patients without baseline MR^4.5^, *n*	79	28	24	85	25	21
MR^4.5^	41.8 (30.8–53.4)	42.9 (24.5–62.8)	45.8 (25.6–67.2)	42.4 (31.7–53.6)	44.0 (24.4–65.1)	42.9 (21.8–66.0)

Evaluable population. To be considered a responder, the patient must have maintenance of baseline response while on treatment (CCyR only) or an improvement from baseline. MMR: *BCR*::*ABL1* IS ≤ 0.1%; MR^4^: *BCR*::*ABL1* IS ≤ 0.01%; MR^4.5^: *BCR*::*ABL1* IS ≤ 0.0032%. Associated two-sided 95% CI based on the exact method by Clopper–Pearson.

*CCyR* complete cytogenic response, *CI* confidence interval, *CML* chronic myeloid leukemia, *CP* chronic phase, *IS* international scale, *mCCI* Charlson Comorbidity Index without age component, *MMR* major molecular response, *MR* molecular response, *Ph* Philadelphia chromosome, *TKI* tyrosine kinase inhibitor.

**Fig. 3:**
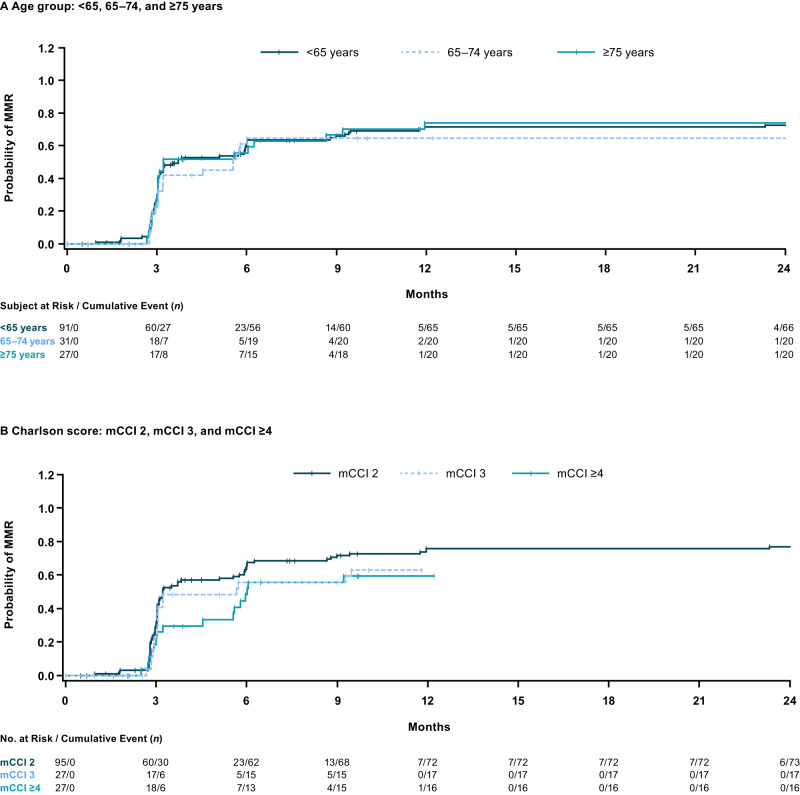
Cumulative incidence of major molecular response in patients with Ph+ CP CML. Patients grouped by (**A**) age and (**B**) comorbidities. CML chronic myeloid leukemia, CP chronic phase, mCCI Charlson Comorbidity Index without age component, MMR major molecular response, Ph Philadelphia chromosome.

In all patients evaluable for molecular response, cumulative MMR rates at any time on treatment were 73.6%, 64.5%, and 74.1% in patients <65, 65–74, and ≥75 years of age, respectively (Fig. 3). Corresponding MR^4^ rates for the three age groups at any time on treatment were 59.3%, 51.6%, and 66.7%, and MR^4.5^ rates were 47.3%, 48.4%, and 51.9%, respectively (Table 2). In patients with mCCI scores 2, 3, and ≥4, cumulative MMR rates at any time on treatment were 77.9%, 63.0%, and 59.3%, respectively (Fig. 3). Corresponding MR^4^ rates for the three comorbidity groups at any time on treatment were 62.1%, 55.6%, and 51.9%, and MR^4.5^ rates were 48.4%, 48.1%, and 48.1%, respectively (Table 2). Among patients without the respective baseline response, respective cumulative MMR, MR^4^, and MR^4.5^ rates at any time on treatment were 66.7%, 52.3%, and 41.8% in patients <65 years of age; 47.6%, 42.3%, and 42.9% in patients 65–74 years of age; and 53.8%, 61.9%, and 45.8% in patients ≥75 years of age (Table 2). Cumulative MMR, MR^4^, and MR^4.5^ rates at any time on treatment in patients without the baseline response were, respectively, 66.7%, 52.9%, and 42.4% in patients with mCCI 2; 46.2%, 54.5%, and 44.0% in patients with mCCI 3; and 46.7%, 45.0%, and 42.9% in patients with mCCI ≥4.

The majority of patients who had dose reductions to 400 mg, 300 mg, or 200 mg QD achieved MMR for the first time or maintained a previous attained MMR after dose reduction (Table 3). Of the patients who remained on ≥500 mg QD, MMR was achieved or maintained in, respectively, 63.2%, 50.0%, and 33.3% of patients <65, 65–74, and ≥75 years of age; and 60.9%, 50.0%, and 33.3% of patients with mCCI 2, 3, and ≥4, respectively.

**Table 3:** Patients with Ph+ CP CML who maintained or attained MMR after dose reduction (by age and comorbidities).

	By age			By comorbidities		
*n*/*N* (%)	<65 years	65–74 years	≥75 years	mCCI 2	mCCI 3	mCCI ≥ 4
No dose reductions (500 mg QD), *n*	19	10	3	23	6	3
MMR, *n* (%)	12 (63.2)	5 (50.0)	1 (33.3)	14 (60.9)	3 (50.0)	1 (33.3)
Dose reduction to 400 mg QD, *n*	23	9	3	24	6	5
MMR, *n* (%)	14 (60.9)	6 (66.7)	2 (66.7)	14 (58.3)	4 (66.7)	4 (80.0)
Dose reduction to 300 mg QD, *n*	28	7	11	23	8	15
MMR, *n* (%)	18 (64.3)	4 (57.1)	9 (81.8)	19 (82.6)	4 (50.0)	8 (53.3)
Dose reduction to 200 mg QD, *n*	22	5	11	27	6	5
MMR, *n* (%)	19 (86.4)	4 (80.0)	9 (81.8)	23 (85.2)	5 (83.3)	4 (80.0)

*CML* chronic myeloid leukemia, *CP* chronic phase, *mCCI* Charlson Comorbidity Index without age component, *MMR* major molecular response, *Ph* Philadelphia chromosome, *QD* once daily.

No patient experienced on-treatment transformation to AP or blast phase CML. Median (range) follow-up for OS was 47.9 (3.6–57.1), 47.9 (2.0–55.0), and 46.1 (0.7–50.7) months in patients <65, 65–74, and ≥75 years of age, respectively. Kaplan–Meier estimated OS rates (95% CI) at 4 years were 100.0% (100.0–100.0) in patients <65 years of age, 68.3% (49.0–81.5) with 10 deaths during the study period in patients 65–74 years of age, and 73.3% (52.0–86.3) with seven deaths in patients ≥75 years of age. Median (range) follow-up for OS was 47.9 (0.7–57.1), 47.6 (3.6–51.7), and 47.2 (2.0–53.0) months in patients with mCCI scores 2, 3, and ≥4, respectively. Kaplan–Meier estimated OS rates (95% CI) at 4 years were 94.7% (87.6–97.7) with five deaths in patients with mCCI 2, 75.5% (53.1–88.3) with six deaths in patients with mCCI 3, and 78.9% (58.9–89.9) with six deaths in patients with mCCI ≥4. In the overall population, two deaths were considered to be related to CML (off-treatment progression to AP/blast phase, *n* = 1; cardiogenic shock, *n* = 1), and none were considered to be treatment-related by the investigators (Supplementary Table 3).

### Safety

**Fig. 4:**
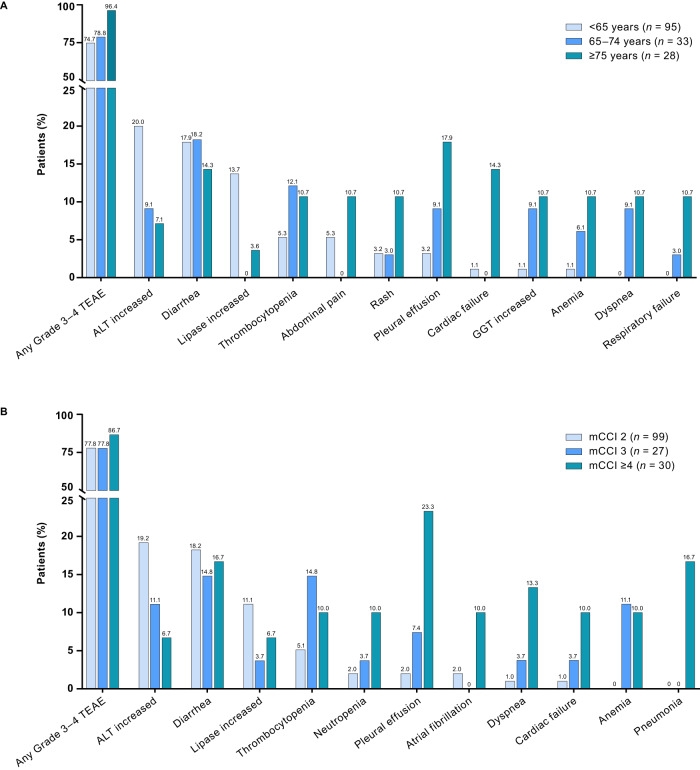
Summary of grade 3–4 TEAEs in patients with Ph+ CP CML. Patients grouped by (**A**) age and (**B**) comorbidities. Includes TEAEs occurring in ≥10% of patients in any subgroup. ALT alanine aminotransferase, CML chronic myeloid leukemia, CP chronic phase, GGT gamma-glutamyl transferase, mCCI Charlson Comorbidity Index without age component, Ph Philadelphia chromosome, TEAE treatment-emergent adverse event.

The incidence of any grade TEAEs was similar between the age groups and between the mCCI risk groups (Supplementary Table 4). Grade 3–4 TEAEs and serious TEAEs, respectively, occurred in 74.7% and 26.3% of patients <65 years of age, 78.8% and 63.6% of patients 65–74 years of age, and 96.4% and 67.9% of patients ≥75 years of age. Respectively, grade 3–4 TEAEs and serious TEAEs occurred in 77.8% and 29.3% of patients with mCCI 2, 77.8% and 48.1% of patients with mCCI 3, and 86.7% and 76.7% of patients with mCCI ≥4. The most frequent grade 3–4 TEAEs by age and mCCI scores are shown in Fig. 4A, B, respectively. Bosutinib was permanently discontinued due to AEs by 22.1%, 39.4%, and 46.4% of patients <65, 65–74, and ≥75 years of age, and 25.3%, 33.3%, and 43.3% of patients with mCCI 2, 3, and ≥4, respectively (Supplementary Table 4). The AEs leading to treatment discontinuation are listed in Supplementary Table 5. Bosutinib dose reductions due to TEAEs occurred in 80.0%, 69.7%, and 89.3% of patients in the three age groups and in 76.8%, 77.8%, and 90.0% of patients with mCCI 2, 3, and ≥4, respectively (Supplementary Table 4).

Medical history and incidences of TEAEs of special interest for the overall population are shown in Supplementary Table 6. Patients with a medical history of liver, myelosuppression, edema, renal, effusion, cardiac, and vascular cluster events were more likely (≥10% increase) to experience the corresponding TEAE event of interest than patients without the relevant medical history.

### Patient-reported outcomes

**Fig. 5:**
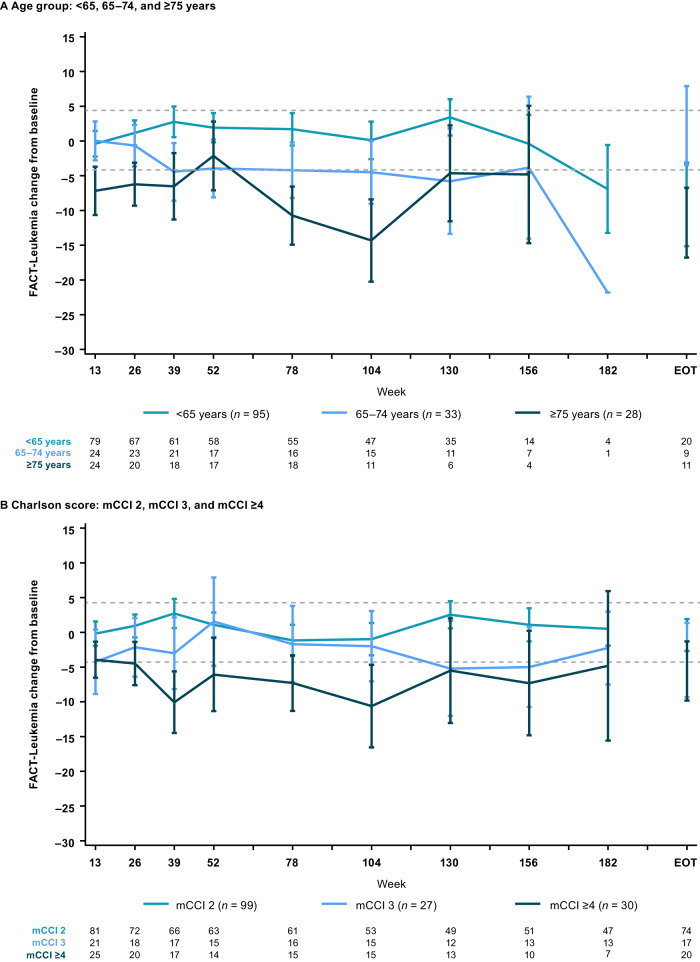
Mean change from baseline on the FACT-Leu leukemia-specific subscale in patients with Ph+ CP CML. Patients grouped by (**A**) age and (**B**) comorbidities. The dashed line indicates the MID (i.e., the change identified as being critically meaningful to a patient). CML chronic myeloid leukemia, CP chronic phase, EOT end of treatment, FACT-Leu Functional Assessment of Cancer Therapy-Leukemia, mCCI Charlson Comorbidity Index without age component, MID minimal important difference, Ph Philadelphia chromosome.

At baseline, mean (standard deviation) FACT-Leu leukemia-specific subscale scores were, respectively, 128.9 (24.7), 131.1 (20.8), and 125.7 (18.9) in patients <65, 65–74, and ≥75 years; and 129.7 (23.1), 123.9 (24.5), and 130.3 (20.6) in patients with mCCI 2, 3 and ≥4. Over the course of treatment, mean FACT-Leu leukemia-specific scores were maintained in patients aged <65 years and in patients with mCCI 2 (Fig. 5). Scores were generally maintained during the course of treatment in patients aged 65–74 years, although there was a decrease at week 182 based on data from one patient. Decreases met the MID at specific timepoints in patients aged ≥75 years, and in patients with mCCI 3 and 4 (Fig. 5).

## Discussion

After ≥3 years of follow-up in the phase 4 BYOND study, a substantial proportion of patients across age and mCCI groups achieved/maintained cytogenetic and molecular responses. Cumulative CCyR and MMR rates were comparable in patients across age groups. Older patients (≥75 years) and those with high comorbidity burden (mCCI ≥4) had higher rates of grade 3/4 and serious TEAEs. There was a trend toward higher rates of treatment discontinuation due to AEs with increasing age and in those patients with a high comorbidity burden. Across age and mCCI groups, dose reductions and interruptions were frequently used to manage AEs. Dose reduction to 400, 300, or 200 mg QD to manage TEAEs did not have an impact on efficacy in any subgroup. This is consistent with a previous study of bosutinib in patients with CP CML resistant/intolerant to prior therapy, which demonstrated that dose reduction did not compromise efficacy while reducing the occurrence of some common TEAEs, such as gastrointestinal TEAEs, even at doses as low as bosutinib 200 mg QD [35].

As expected in patients receiving TKIs, the estimated OS rate at 48 months was high (>68%) in each subgroup [36]. In the overall population, only two deaths were due to CML, and no deaths were related to bosutinib treatment (as determined by investigators). Determining a significant prognostic factor for survival may be difficult given the low number of death events seen in this study. Previous studies in patients with CML treated with TKIs have shown that higher CCI is associated with reduced OS, with patients dying from their comorbidities rather than from CML [15–20].

Patients with a medical history of liver, myelosuppression, edema, renal, effusion, cardiac, and vascular cluster events were more likely to experience the corresponding TEAE event of interest than those patients without the relevant medical history. Therefore, proactive management and monitoring may be warranted in patients with these preexisting conditions. These results are generally consistent with what has previously been reported for bosutinib treatment [37].

Health-related quality of life was maintained from baseline throughout treatment for patients aged <65 and 65–74 years and patients with mCCI 2. A meaningful reduction in health-related quality of life was seen at multiple timepoints on treatment in patients aged ≥75 years, and patients with mCCI 3 and 4. A potential reason for this is that the increase in grade 3–4 and serious TEAEs observed in these subgroups of patients adversely affected health-related quality of life, as has been previously reported in patients receiving TKIs [38, 39].

Together these results suggest that while a substantial proportion of patients maintained/achieved cytogenetic and molecular responses with bosutinib across age groups and mCCI scores, both comorbidities and age are important factors to consider during bosutinib treatment. Older patients and those with a high comorbidity burden are more likely to experience more severe TEAEs, which can also impact health-related quality of life. Age, comorbidities, and risk factors, as well as the safety profile and schedule of administration of TKIs, should be considered when selecting the most appropriate TKI for the treatment of patients with previously treated CML. Patients should be monitored throughout treatment, not only for response but also for health conditions, whether they were preexisting or developed during therapy [40]. Guidelines for the selection of TKIs in patients with CML and the management of AEs occurring with bosutinib treatment have been published [37, 41].

The phase 2 Bosutinib in Elderly Chronic Myeloid Leukemia (BEST) and Bosutinib Dose Optimization (BODO) studies investigated dose optimization strategies to minimize the severity and incidence of AEs [42, 43]. The BEST study in elderly patients (aged 60–90 years) with CP CML resistant/intolerant to first-line TKIs demonstrated efficacy when using lower doses of bosutinib [43]. Treatment was initiated using 200 mg QD and increased (maximum of 400 mg QD) according to the molecular response to find the minimum effective dose. In the final results, 79% of patients remained on treatment with bosutinib (88% of patients receiving ≤300 mg QD), with 65% of patients achieving or maintaining MMR. The BODO study aimed to evaluate whether a bosutinib step-in dosing regimen (starting dose 300 mg QD) could decrease gastrointestinal toxicity while maintaining optimal efficacy in patients with CML after failure or intolerance to second-generation TKIs [42]. While the study failed to demonstrate a reduction in gastrointestinal toxicity due to incomplete recruitment, 19 out of 30 patients (63%) achieved MMR or better with the step-in dosing regimen. In a real-world study of patients aged >65 years intolerant or resistant to previous TKIs, the majority (75.2%) of patients received bosutinib at a starting dose <500 mg QD [44]. Despite the lower starting doses, bosutinib was effective with a manageable safety profile in elderly patients with multiple comorbidities. Together these studies suggest that bosutinib dose modifications are an important consideration for the management of AEs, particularly in older patients and those with multiple comorbidities.

Although comparisons between studies are difficult due to differences in methodologies and patient populations, the results reported here for bosutinib appear comparable to those with other second-generation TKIs. In a retrospective study of patients with resistance or intolerance to imatinib who received dasatinib, stratification by Charlson Comorbidity Index was able to identify patients at risk of major toxicities such as pleural effusion [22]. A subanalysis of the ENEST1st study, evaluating the impact of age on the efficacy and safety nilotinib in patients with newly diagnosed CML, showed that age did not have an impact on deep molecular response rates while slight differences in the incidence of AEs were observed [30].

In conclusion, the findings of this study further support bosutinib use for patients with Ph+ CP CML across age groups and mCCI scores. Age and mCCI stratification may enable the identification of patients who are at higher risk of developing TEAEs and may require more careful monitoring in clinical practice.

Information about this study in a plain language format is available in the supplementary materials.

### Supplementary information

Supplemental Material and Plain Language Summary

## Data Availability

Upon request, and subject to review, Pfizer will provide the data that support the findings of this study. Subject to certain criteria, conditions and exceptions, Pfizer may also provide access to the related individual de-identified participant data. See https://www.pfizer.com/science/clinical-trials/trial-data-and-results for more information.
