# Supplementary material for: Impact of age and comorbidities on the efficacy and tolerability of bosutinib in previously treated patients with chronic myeloid leukemia: results from the phase 4 BYOND study
Source: Leukemia. 2023 Nov 25;38(1):126–35. doi: 10.1038/s41375-023-02080-y (PMC10776383; doi:10.1038/s41375-023-02080-y)
Supplement: Supplementary file 1 — Supplemental Material and Plain Language Summary [file 41375_2023_2080_MOESM1_ESM.pdf]

SUPPLEMENTARY MATERIALS

Supplementary Methods

Analysis of Comorbidities

Comorbidities were analyzed using the Charlson Comorbidity Index without the age component (mCCI) [1]. Relevant comorbid conditions are weighted from 1 to 6 points. Comorbid conditions included in the mCCI:

1 point	2 points	3 points	6 points
• Myocardial infarction<br>• Congestive heart failure<br>• Peripheral vascular disease<br>• Cerebrovascular disease<br>• Dementia<br>• Chronic pulmonary disease<br>• Connective tissue disease<br>• Peptic ulcer disease<br>• Mild liver disease<br>• Diabetes	• Hemiplegia<br>• Diabetes with complications<br>• Renal disease<br>• Leukemia<br>• Lymphoma<br>• Tumor without metastases	• Moderate/severe liver disease	• HIV/AIDS<br>• Metastatic solid tumor

Safety assessments

Frequency and characteristics of adverse events (AEs) of special interest were analyzed by selecting Medical Dictionary for Regulatory Activities (MedDRA) system organ class high-level group, high-level and preferred terms (PT), and standardized MedDRA queries to generate treatment-

emergent adverse event (TEAE) clusters. MedDRA terms included in the TEAE clusters were as follows:

- **Cardiac.** *High-level group terms (HLGTs):* cardiac arrhythmias, heart failures, pericardial disorders; *PT:* cardiac death, sudden cardiac death, sudden death, ejection fraction decreased; *Standardized MedDRA query (SMQ):* torsade de pointes / QT prolongation (narrow).
- **Edema.** *PT:* contains edema or weight increased.
- **Effusion.** *PT:* pericardial effusion or pleural effusion.
- **Gastrointestinal.** *PT:* nausea, regurgitation, retching, vomiting, vomiting projectile, diarrhea, defecation urgency, frequent bowel movements, gastrointestinal hypermotility.
- **Hypertension.** *HLGT:* vascular hypertension disorders; *PT:* blood pressure (BP) abnormal, BP ambulatory abnormal, BP ambulatory increased, BP diastolic abnormal, BP diastolic increased, BP increased, BP systolic abnormal, BP systolic increased.
- **Liver.** *Sub-SMQ (Narrow):* cholestasis and jaundice of hepatic origin; hepatic failure, fibrosis, and cirrhosis and other liver damage-related conditions; hepatitis, noninfectious; *PT:* alanine aminotransferase (ALT) abnormal, ALT increased, aspartate aminotransferase (AST) abnormal, AST increased, bilirubin conjugated abnormal, bilirubin conjugated increased, blood bilirubin abnormal, blood bilirubin increased, blood bilirubin unconjugated increased, hepatic enzyme abnormal, hepatic enzyme increased, hepatic function abnormal, hyperbilirubinemia, hypertransaminasemia, liver function test abnormal, liver function test increased, transaminase abnormal, transaminase increased, blood alkaline phosphatase abnormal, blood alkaline phosphatase increased.
- **Myelosuppression.** *SMQ (Narrow):* hematopoietic cytopenias affecting more than one type of blood cell, hematopoietic erythropenia, hematopoietic leukopenia, hematopoietic thrombocytopenia; *PT:* hematocrit decreased, hemoglobin decreased, hematotoxicity, anemia.
- **Rash.** *Higher-level terms (HLT)*: rashes, eruptions and exanthemas not elsewhere classified (NEC), erythemas, acnes, dermatitis and eczema.
- **Renal**. *HLT:* renal failure and impairment; *PT:* blood creatinine abnormal, blood creatinine increased, creatinine renal clearance abnormal, creatinine renal clearance decreased, glomerular filtration rate abnormal, glomerular filtration rate decreased.

- **Vascular** includes MedDRA terms for cardiovascular, cerebrovascular, and peripheral vascular TEAEs:
  - ***Cardiovascular*****:** *HLGT:* coronary artery disorders; *HLT:* arterial therapeutic procedures (excluding aortic), vascular imaging procedures NEC, vascular therapeutic procedures NEC; *PT:* transcatheter arterial chemoembolization.
  - ***Cerebrovascular*****:** *HLT:* central nervous system (CNS) hemorrhages and cerebrovascular accidents, CNS vascular disorders NEC, transient cerebrovascular events.
  - ***Peripheral vascular****: HLGT:* arteriosclerosis, stenosis, vascular insufficiency and necrosis, embolism and thrombosis; *HLT:* non–site-specific vascular disorders NEC, peripheral vascular disorders NEC (excluding PTs flushing and hot flush); *PT:* intestinal ischemia.

REFERENCES

1. Hall WH, Ramachandran R, Narayan S, Jani AB, Vijayakumar S. An electronic application for rapidly calculating Charlson comorbidity score. BMC Cancer. 2004;4:94.

Supplementary Table 1. Treatment and study summary in patients with Ph+ CP CML by age and comorbidities.

	**By age**			**By comorbidities**		
	**<65 years** *n* = 95	**65−74 years** *n* = 33	**≥75 years** *n* = 28	**mCCI 2** *n* = 99	**mCCI 3** *n* = 27	**mCCI ≥4** *n* = 30
Median (range) duration of treatment, months	47.6 (0.4–48.9)	34.7 (1.6–50.1)	27.1 (0.2–48.4)	46.7 (0.2–50.1)	44.9 (0.8–48.7)	24.8 (1.6–48.4)
Median (range) dose intensity, mg/day	320.9 (145.0–560.6)	319.3 (79.7–500.0)	264.7 (98.4–520.4)	320.8 (125.0–560.6)	296.1 (98.4–500.0)	300.3 (79.7–496.6)
Discontinued treatment, *n* (%)	38 (40.0)	22 (66.7)	21 (75.0)	44 (44.4)	15 (55.6)	22 (73.3)
AE	20 (21.1)	11 (33.3)	11 (39.3)	24 (24.2)	6 (22.2)	12 (40.0)
Related to study treatment	19 (20.0)	8 (24.2)	5 (17.9)	17 (17.2)	5 (18.5)	10 (33.3)
Unrelated to study treatment	1 (1.1)	3 (9.1)	6 (21.4)	7 (7.1)	1 (3.7)	2 (6.7)
Lack of efficacy	4 (4.2)	2 (6.1)	3 (10.7)	4 (4.0)	1 (3.7)	4 (13.3)
Patient died	0	2 (6.1)	2 (7.1)	0	3 (11.1)	1 (3.3)
Lost to follow-up	1 (1.1)	0	0	1 (1.0)	0	0
Noncompliance	2 (2.1)	3 (9.1)	1 (3.6)	4 (4.0)	0	2 (6.7)
Other	5 (5.3)	2 (6.1)	2 (7.1)	6 (6.1)	3 (11.1)	0
Protocol violation	1 (1.1)	1 (3.0)	1 (3.6)	1 (1.0)	0	2 (6.7)
Patient request	5 (5.3)	0	1 (3.6)	4 (4.0)	2 (7.4)	0
Completed study, *n* (%)	74 (77.9)	19 (57.6)	14 (50.0)	74 (74.7)	13 (48.1)	20 (66.7)
Study terminated by Sponsor, *n* (%)	10 (10.5)	3 (9.1)	2 (7.1)	9 (9.1)	4 (14.8)	2 (6.7)

AE adverse event, CML chronic myeloid leukemia, CP chronic phase, mCCI Charlson Comorbidity Index without age component, Ph Philadelphia chromosome.

Supplementary Table 2. Summary of treatment discontinuation over time in patients with Ph+ CP CML by age and comorbidities.

	**Year 1**	**Year 2**	**Year 3**	**Year 4+**
**By age**				
**<65 years (*n* = 95)**				
Discontinued treatment, *n* (%)	29 (30.5)	4 (4.2)	2 (2.1)	3 (3.2)
AE	18 (18.9)	1 (1.1)	0	1 (1.1)
Lack of efficacy	3 (3.2)	0	0	1 (1.1)
Lost to follow-up	0	1 (1.1)	0	0
Noncompliance	1 (1.1)	1 (1.1)	0	0
Other	2 (2.1)	1 (1.1)	2 (2.1)	0
Protocol violation	1 (1.1)	0	0	0
Patient request	4 (4.2)	0	0	1 (1.1)
**65−74 years (*n* = 33)**				
Discontinued treatment, *n* (%)	12 (36.4)	4 (12.1)	2 (6.1)	4 (12.1)
AE	6 (18.2)	3 (9.1)	1 (3.0)	1 (3.0)
Lack of efficacy	2 (6.1)	0	0	0
Patient died	1 (3.0)	0	0	1 (3.0)
Noncompliance	0	1 (3.0)	1 (3.0)	1 (3.0)
Other	2 (6.1)	0	0	1 (3.0)
Protocol violation	1 (3.0)	0	0	0
**≥75 years (*n* = 28)**				
Discontinued treatment, *n* (%)	8 (28.6)	3 (10.7)	7 (25.0)	3 (10.7)
AE	4 (14.3)	2 (7.1)	5 (27.9)	0
Lack of efficacy	3 (10.7)	0	0	0
Patient died	0	0	1 (3.6)	1 (3.6)
Noncompliance	0	1 (3.6)	0	0
Other	0	0	0	2 (7.1)
Protocol violation	0	0	1 (3.6)	0
Patient request	1 (3.6)	0	0	0
**By comorbidities**				
**mCCI 2 (*n* = 99)**				

Discontinued treatment, *n* (%)	27 (27.3)	9 (9.1)	4 (4.0)	4 (4.0)
AE	16 (16.2)	5 (5.1)	1 (1.0)	2 (2.0)
Lack of efficacy	3 (3.0)	0	0	1 (1.)
Lost to follow-up	0	1 (1.0)	0	0
Noncompliance	1 (1.0)	2 (2.0)	1 (1.0)	0
Other	3 (3.0)	1 (1.0)	2 (2.0)	0
Protocol violation	1 (1.0)	0	0	0
Patient request	3 (3.0)	0	0	1 (1.0)
**mCCI 3 (*n* = 27)**				
Discontinued treatment, *n* (%)	10 (37.0)	0	2 (7.4)	3 (11.1)
AE	5 (18.5)	0	1 (3.7)	0
Lack of efficacy	1 (3.7)	0	0	0
Patient died	1 (3.7)	0	1 (3.7)	1 (3.7)
Other	1 (3.7)	0	0	2 (7.4)
Patient request	2 (7.4)	0	0	0
**mCCI ≥4 (*n* = 30)**				
Discontinued treatment, *n* (%)	12 (40.0)	2 (6.7)	5 (16.7)	3 (10.0)
AE	7 (23.3)	1 (3.3)	4 (13.3)	0
Lack of efficacy	4 (13.3)	0	0	0
Patient died	0	0	0	1 (3.3)
Noncompliance	0	1 (3.3)	0	1 (3.3)
Other	0	0	0	1 (3.3)
Protocol violation	1 (3.3)	0	1 (3.3)	0

AE adverse event

Supplementary Table 3. Listing of deaths

	Cause of death	Study phase
Patient 1	Acute kidney injury	On-treatment
Patient 2	Heart failure	On-treatment
Patient 3	Prostate adenocarcinoma	On-treatment
Patient 4	Disease under study	On-treatment
Patient 5	Multi-organ failure	On-treatment
Patient 6	Heart failure	On-treatment
Patient 7	New cancer diagnosis of lymphoma	On-treatment
Patient 8	Exitus litalis	Follow-up
Patient 9	Unknown	Follow-up
Patient 10	Disease under study	Follow-up
Patient 11	Chronic bridenileus	Follow-up
Patient 12	Central nervous system bleeding	Follow-up
Patient 13	Pancreatic cancer with liver metastasis	Follow-up
Patient 14	Colon carcinoma	Follow-up
Patient 15	Sepsis	Follow-up
Patient 16	Small cell lung cancer	Follow-up
Patient 17	Cerebral tumor	Follow-up

Supplementary Table 4. Summary of adverse events in patients with Ph+ CP CML by age and comorbidities.

TEAE, *n* (%)	By age			By comorbidities		
	**<65 years** *n* = 95	**65–74 years** *n* = 33	**≥75 years** *n* = 28	**mCCI 2** *n* = 99	**mCCI 3** *n* = 27	**mCCI ≥4** *n* = 30
Any grade TEAE	95 (100.0)	32 (97.0)	28 (100.0)	98 (99.0)	27 (100.0)	30 (100.0)
Grade 3–4 TEAEs	71 (74.7)	26 (78.8)	27 (96.4)	77 (77.8)	21 (77.8)	26 (86.7)
Serious AEs	25 (26.3)	21 (63.6)	19 (67.9)	29 (29.3)	13 (48.1)	23 (76.7)
AEs leading to treatment discontinuation	21 (22.1)	13 (39.4)	13 (46.4)	25 (25.3)	9 (33.3)	13 (43.3)
TEAEs leading to dose reduction	76 (80.0)	23 (69.7)	25 (89.3)	76 (76.8)	21 (77.8)	27 (90.0)
TEAEs leading to temporary stop	70 (73.7)	23 (69.7)	26 (92.9)	70 (70.7)	22 (81.5)	27 (90.0)
TEAEs leading to death	0	2 (6.1)	5 (17.9)	3 (3.0)	3 (11.1)	1 (3.3)

TEAEs were defined as any event increasing in severity from baseline or any new event starting during bosutinib therapy or within 28 days of the last dose of study drug. Classifications of AEs are based on the Medical Dictionary for Regulatory Activities (MedDRA version 21.1). AEs adverse events, CML chronic myeloid leukemia, CP chronic phase, mCCI Charlson Comorbidity Index without age component, Ph Philadelphia chromosome, TEAE treatment-emergent adverse event.

Supplementary Table 5. AEs leading to treatment discontinuation in patients with Ph+ CP CML by age and comorbidities.

AE, *n* (%)	By age			By comorbidities		
	**<65 years** *n* = 95	**65−74 years** *n* = 33	**≥75 years** *n* = 28	**mCCI 2** *n* = 99	**mCCI 3** *n* = 27	**mCCI ≥4** *n* = 30
Any AE	21 (22.1)	13 (39.4)	13 (46.4)	25 (25.3)	9 (33.3)	13 (43.3)
Abdominal pain	1 (1.1)	0	0	1 (1.0)	0	0
Acute kidney injury	0	1 (3.0)	1 (3.6)	0	1 (3.7)	1 (3.3)
Alanine aminotransferase increased	6 (6.3)	2 (6.1)	0	7 (7.1)	1 (3.7)	0
Anemia	0	0	1 (3.6)	0	1 (3.7)	0
Aspartate aminotransferase increased	4 (4.2)	0	0	4 (4.0)	0	0
Aortic aneurysm	0	0	1 (3.6)	0	0	1 (3.3)
Aortic valve incompetence	0	0	1 (3.6)	0	0	1 (3.3)
Brain neoplasm	0	1 (3.0)	0	1 (1.0)	0	0
Cardiac failure	0	1 (3.0)	2 (7.1)	0	1 (3.7)	2 (6.7)
Cardiogenic shock	0	1 (3.0)	0	0	1 (3.7)	0
Decreased appetite	1 (1.1)	0	0	1 (1.0)	0	0
Diarrhea	1 (1.1)	1 (3.0)	1 (3.6)	1 (1.0)	1 (3.7)	1 (3.3)
Dyspnea	0	0	1 (3.6)	1 (1.0)	0	0
Edema peripheral	0	1 (3.0)	0	1 (1.0)	0	0
Fatigue	1 (1.1)	0	0	1 (1.0)	0	0
Fluid retention	1 (1.1)	0	0	0	0	1 (3.3)
Ileus	0	1 (3.0)	0	0	0	1 (3.3)
Lipase increased	1 (1.1)	0	0	0	0	1 (3.3)
Lymphoma	0	0	1 (3.6)	1 (1.0)	0	1 (3.3)
Nausea	2 (2.1)	1 (3.0)	0	2 (2.0)	0	1 (3.3)
Neutropenia	2 (2.1)	0	0	1 (1.0)	0	1 (3.3)

Pancreatitis	0	1 (3.0)	0	0	0	1 (3.3)
Pancreatic carcinoma	0	1 (3.0)	0	1 (1.0)	0	0
Pericardial effusion	0	1 (3.0)	0	0	0	1 (3.3)
Peripheral ischemia	0	0	1 (3.6)	0	0	1 (3.3)
Pleural effusion	1 (1.1)	1 (3.0)	1 (3.6)	1 (1.0)	0	2 (6.7)
Pneumonia	0	1 (3.0)	0	0	0	1 (3.3)
Prostate cancer	0	0	1 (3.6)	1 (1.0)	0	0
Pulmonary hypertension	2 (2.1)	0	0	1 (1.0)	1 (3.7)	0
Pseudomembranous colitis	0	0	1 (3.6)	0	1 (3.7)	0
Rash	1 (1.1)	1 (3.0)	0	1 (1.0)	1 (3.7)	0
Rash maculo-papular	1 (1.1)	0	0	1 (1.0)	0	0
Rash papular	1 (1.1)	0	0	1 (1.0)	0	0
Subdural hematoma	1 (1.1)	0	0	1 (1.0)	0	0
Syncope	0	0	1 (3.6)	1 (1.0)	0	0
Ventricular dysfunction	0	0	1 (3.6)	0	0	1 (3.3)
Vomiting	2 (2.1)	0	0	2 (2.0)	0	0

AE adverse event, ALT alanine aminotransferase, AST aspartate aminotransferase, CP chronic phase, CML chronic myeloid leukemia, Ph Philadelphia chromosome, mCCI Charlson Comorbidity Index without age component, Ph Philadelphia chromosome.

Supplementary Table 6. Medical History by TEAE Cluster in Patients with Ph+ CP CML

***n*/*N* (%)**	**Total Ph+ CP CML (*N* = 156)**
**Gastrointestinal cluster**	
Patients with events	145/156 (92.9)
Patients with medical history of any gastrointestinal cluster event	22/156 (14.1)
Patients with events and with a medical history of any gastrointestinal cluster event	22/22 (100.0)
Patients with events and without a medical history of gastrointestinal cluster event	123/134 (91.8)
**Liver cluster**	
Patients with events	49/156 (31.4)
Patients with medical history of any liver cluster event	13/156 (8.3)
Patients with events and with a medical history of any liver cluster event	8/13 (61.5)
Patients with events and without a medical history of any liver cluster event	41/143 (28.7)
**Rash cluster**	
Patients with events	49/156 (31.4)
Patients with medical history of any rash cluster event	29/156 (18.6)
Patients with events and with a medical history of any rash cluster event	11/29 (37.9)
Patients with events and without a medical history of any rash cluster event	38/127 (29.9)
**Myelosuppression cluster**	
Total patients with TEAEs	40/156 (25.6)
Patients with medical history of any myelosuppression cluster event	30/156 (19.2)
Patients with events and with a medical history of any myelosuppression cluster event	16/30 (53.3)

Patients with events and without a medical history of any myelosuppression cluster event	24/126 (19.0)
**Edema cluster**	
Total patients with TEAEs	39/156 (25.0)
Patients with medical history of any edema cluster event	35/156 (22.4)
Patients with events and with a medical history of any edema cluster event	13/35 (37.1)
Patients with events and without a medical history of any edema cluster event	26/121 (21.5)
**Renal cluster**	
Total patients with TEAEs	37/156 (23.7)
Patients with medical history of any renal cluster event	14/156 (9.0)
Patients with events and with a medical history of any renal cluster event	8/14 (57.1)
Patients with events and without a medical history of any renal cluster event	29/142 (20.4)
**Effusion cluster**	
Total patients with TEAEs	34/156 (21.8)
Patients with medical history of any effusion cluster event	43/156 (27.6)
Patients with events and with a medical history of any effusion cluster event	17/43 (39.5)
Patients with events and without a medical history of any effusion cluster event	17/113 (15.0)
**Cardiac cluster**	
Total patients with TEAEs	30/156 (19.2)
Patients with medical history of any cardiac cluster event	34/156 (21.8)
Patients with events and with a medical history of any cardiac cluster event	11/34 (32.4)
Patients with events and without a medical history of any cardiac cluster event	19/122 (15.6)
**Vascular cluster**	

Total patients with TEAEs	20/156 (12.8)
Patients with medical history of any vascular cluster event	47/156 (30.1)
Patients with events and with a medical history of any vascular cluster event	15/47 (31.2)
Patients with events and without a medical history of any vascular cluster event	5/109 (4.6)
**Hypertension cluster**	
Total patients with TEAEs	13/156 (8.3)
Patients with medical history of any hypertension cluster event	72/156 (46.2)
Patients with events and with a medical history of any hypertension cluster event	8/72 (11.1)
Patients with events and without a medical history of any hypertension cluster event	5/84 (6.0)

CP chronic phase, CML chronic myeloid leukemia, Ph Philadelphia chromosome, TEAE treatment-emergent adverse event.

Plain Language Summary

Please note that this summary only contains information from the full scientific article:

View Scientific Article

The impact of additional medical conditions and age on the efficacy and safety of bosutinib in people with chronic myeloid leukemia

Bosutinib
<boh-SOO-tih-nib>

Chronic myeloid leukemia
<KRAH-nik MY-eh-loyd loo-KEE-mee-uh>

Dasatinib
<da-SA-tih-nib>

Imatinib
<ih-MA-tih-nib>

Nilotinib
<nye-LOH-tih-nib>

Tyrosine kinase inhibitor
<TY-ruh-seen KY-nays in-HIH-bih-ter>

Date of summary: November 2023

Study number: NCT02228382

Study start date: November 2014

Study end date: October 2020

The full title of this article: Impact of Age and Comorbidities on the Efficacy and Tolerability of Bosutinib in Previously Treated Patients with Chronic Myeloid Leukemia: Results from the Phase 4 BYOND Study

Key takeaways

- In this study, bosutinib worked for most people regardless of their age or if they had additional medical conditions
- Older people or those with additional medical conditions may experience severe side effects more often, and be more likely to stop taking bosutinib because of these side effects

The purpose of this plain language summary is to help you to understand the findings from recent research.

- Bosutinib is approved to treat the condition under study that is discussed in this summary
- This summary reports the results of a single study. The results of this study may differ from those of other studies. Health professionals should make treatment decisions based on all available evidence, not on the results of a single study

More information can be found in the scientific article of this study, which you can access here: View Scientific Article

Additional information

More information can be found in the scientific article of this study, which you can access here:

View Scientific Article

For more information on clinical studies in general, please visit:

https://www.clinicaltrials.gov/ct2/about-studies/learn

https://www.cancerresearchuk.org/about-cancer/find-a-clinical-trial/what-clinical-trials-are

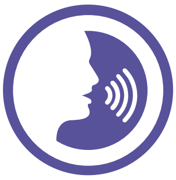

Bosutinib
<boh-SOO-tih-nib>

Chronic myeloid leukemia
<KRAH-nik MY-eh-loyd loo-KEE-mee-uh>

Dasatinib
<da-SA-tih-nib>

Imatinib
<ih-MA-tih-nib>

Nilotinib
<nye-LOH-tih-nib>

Tyrosine kinase inhibitor
<TY-ruh-seen KY-nays in-HIH-bih-ter>

What did this study look at?

- Chronic myeloid leukemia (CML for short) is a type of cancer that affects white blood cells. Chronic means it tends to progress slowly over many years
  - CML is caused by the formation of an abnormal fusion gene called BCR-ABL
  - Genes are segments of DNA and are found in structures called chromosomes. They are found within each cell of the body
    - DNA is a molecule in a person's cells that tells the cells how to work. Chromosomes are bundles of DNA
  - A fusion gene is formed when pieces of two different chromosomes break off and change places. This creates a new gene called BCR-ABL
  - The BCR-ABL gene is found in a chromosome called the Philadelphia chromosome. It is present in some types of leukemia cancer cells
- Bosutinib is a type of medicine known as a tyrosine kinase inhibitor (TKI for short)
  - Tyrosine kinases are proteins in the body that control how cells grow and divide
  - The BCR-ABL gene makes a tyrosine kinase that is more active than normal and makes leukemia cells grow faster than healthy cells
  - Bosutinib works by blocking this more active tyrosine kinase in the leukemia cells, causing those cells to die
- In this study, people with CML who had already received between one and three previous TKI treatments (such as imatinib, dasatinib, or nilotinib) took bosutinib
- These people stopped their previous treatments because:
  - their CML was no longer responding (also known as resistant) to treatment, or
  - they could no longer tolerate their treatment due to side effects (also known as intolerant). A side effect is something (expected or unexpected) that you feel was caused by a medicine or treatment you take
- This summary looks at whether a person's age or having additional medical conditions at the start of taking bosutinib influences how well it works (researchers call this efficacy) and how safe it is

Where is this study in the drug development timeline?

- This was a phase 4 trial, which is a type of clinical trial that looks for further information about a treatment after it has been approved and is used as a treatment

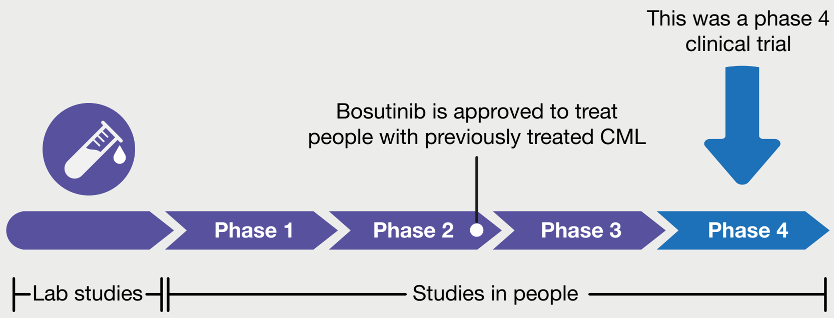

Additional information

More information can be found in the scientific article of this study, which you can access here:

View Scientific Article

For more information on clinical studies in general, please visit:

https://www.clinicaltrials.gov/ct2/about-studies/learn

https://www.cancerresearchuk.org/about-cancer/find-a-clinical-trial/what-clinical-trials-are

Bosutinib
<boh-SOO-tih-nib>

Chronic myeloid leukemia
<KRAH-nik MY-eh-loyd loo-KEE-mee-uh>

Dasatinib
<da-SA-tih-nib>

Imatinib
<ih-MA-tih-nib>

Nilotinib
<nye-LOH-tih-nib>

Tyrosine kinase inhibitor
<TY-ruh-seen KY-nays in-HIH-bih-ter>

Additional information

More information can be found in the scientific article of this study, which you can access here:

View Scientific Article

For more information on clinical studies in general, please visit:

https://www.clinicaltrials.gov/ct2/about-studies/learn

https://www.cancerresearchuk.org/about-cancer/find-a-clinical-trial/what-clinical-trials-are

Who took part in this study?

- Researchers grouped people with CML based on their Charlson Comorbidity Score (CCI score for short) at the start of treatment
  - Their CCI score was determined by the number and severity of any additional medical conditions they had
- Only certain medical conditions (such as liver disease and heart failure) that could potentially impact how long a person lives were included
  - People with a low CCI score had CML only. People with a moderate or high CCI score had additional medical conditions
  - People with a high CCI score had more medical conditions, or their additional medical conditions were more severe, compared to people with a moderate CCI score

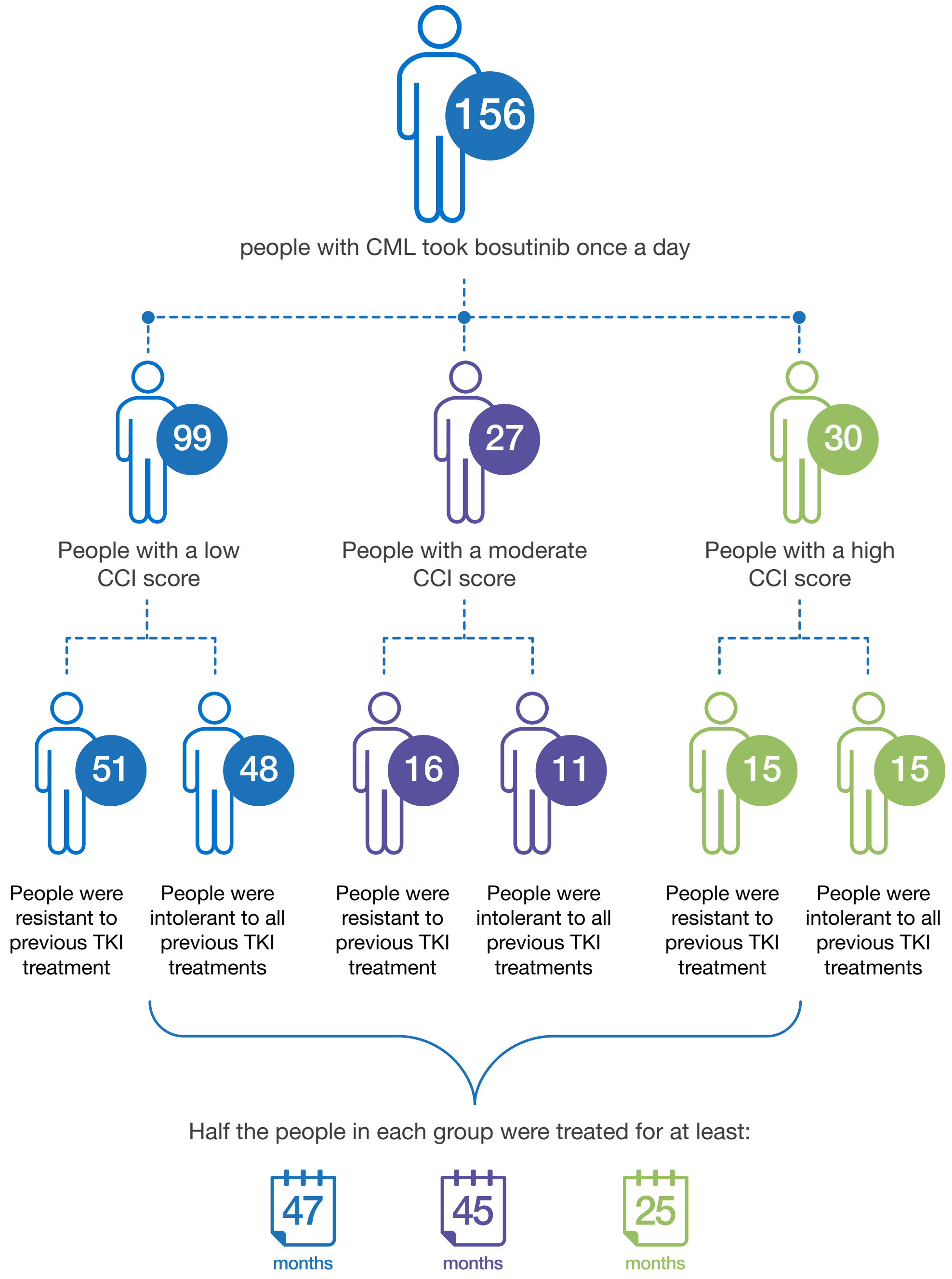

- They also grouped people with CML by their age

Bosutinib
<boh-SOO-tih-nib>

Chronic myeloid leukemia
<KRAH-nik MY-eh-loyd loo-KEE-mee-uh>

Dasatinib
<da-SA-tih-nib>

Imatinib
<ih-MA-tih-nib>

Nilotinib
<nye-LOH-tih-nib>

Tyrosine kinase inhibitor
<TY-ruh-seen KY-nays in-HIH-bih-ter>

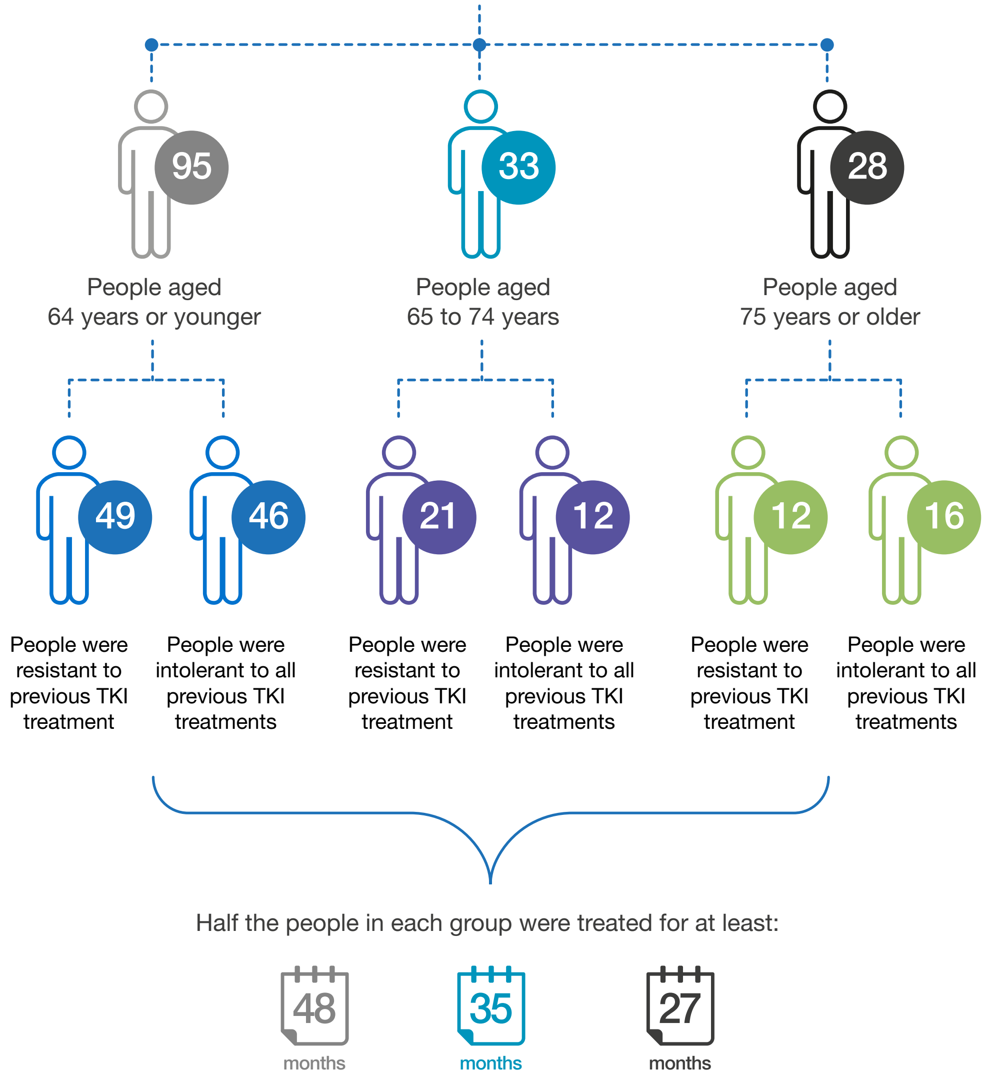

- From the start of the study, people with CML were monitored for up to 4 years

Additional information

More information can be found in the scientific article of this study, which you can access here:

View Scientific Article

For more information on clinical studies in general, please visit:

https://www.clinicaltrials.gov/ct2/about-studies/learn

https://www.cancerresearchuk.org/about-cancer/find-a-clinical-trial/what-clinical-trials-are

Bosutinib
<boh-SOO-tih-nib>

Chronic myeloid leukemia
<KRAH-nik MY-eh-loyd loo-KEE-mee-uh>

Dasatinib
<da-SA-tih-nib>

Imatinib
<ih-MA-tih-nib>

Nilotinib
<nye-LOH-tih-nib>

Tyrosine kinase inhibitor
<TY-ruh-seen KY-nays in-HIH-bih-ter>

What were the results of the study?

Efficacy

- Researchers looked at whether treatment with bosutinib lowered:
  - the number of cells with Philadelphia chromosome in the bone marrow, and
  - the level of the BCR-ABL gene in the blood
- People who respond well to treatment have fewer cells with the Philadelphia chromosome and lower BCR-ABL levels

Following treatment with bosutinib, the proportion of people who had:

	People with a low CCI score	People with a moderate CCI score	People with a high CCI score
no cells with the Philadelphia chromosome (researchers call this a complete cytogenetic response or CCyR for short)	88 in 100	72 in 100	68 in 100
**low** levels of the BCR-ABL gene (researchers call this a major molecular response or MMR for short)	78 in 100	63 in 100	59 in 100
**very low** levels of the BCR-ABL gene (researchers call this a molecular response 4 or MR^4 for short)	62 in 100	56 in 100	52 in 100
**extremely low** levels of the BCR-ABL gene (researchers call this a molecular response 4.5 or $MR^{4.5}$ for short)	48 in 100	48 in 100	48 in 100

	People aged 64 years or younger	People aged 65 to 74 years	People aged 75 years or older
no cells with the Philadelphia chromosome (researchers call this a complete cytogenetic response or CCyR for short)	86 in 100	67 in 100	83 in 100
**low** levels of the BCR-ABL gene (researchers call this a major molecular response or MMR for short)	74 in 100	65 in 100	74 in 100
**very low** levels of the BCR-ABL gene (researchers call this a molecular response 4 or MR^4 for short)	59 in 100	52 in 100	67 in 100
**extremely low** levels of the BCR-ABL gene (researchers call this a molecular response 4.5 or $MR^{4.5}$ for short)	47 in 100	48 in 100	52 in 100

Additional information

More information can be found in the scientific article of this study, which you can access here:

View Scientific Article

For more information on clinical studies in general, please visit:

https://www.clinicaltrials.gov/ct2/about-studies/learn

https://www.cancerresearchuk.org/about-cancer/find-a-clinical-trial/what-clinical-trials-are

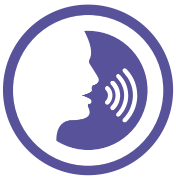

Bosutinib
<boh-SOO-tih-nib>

Chronic myeloid leukemia
<KRAH-nik MY-eh-loyd loo-KEE-mee-uh>

Dasatinib
<da-SA-tih-nib>

Imatinib
<ih-MA-tih-nib>

Nilotinib
<nye-LOH-tih-nib>

Tyrosine kinase inhibitor
<TY-ruh-seen KY-nays in-HIH-bih-ter>

- Researchers also looked at how long people lived after starting bosutinib treatment

The likelihood that a person is alive after 4 years:

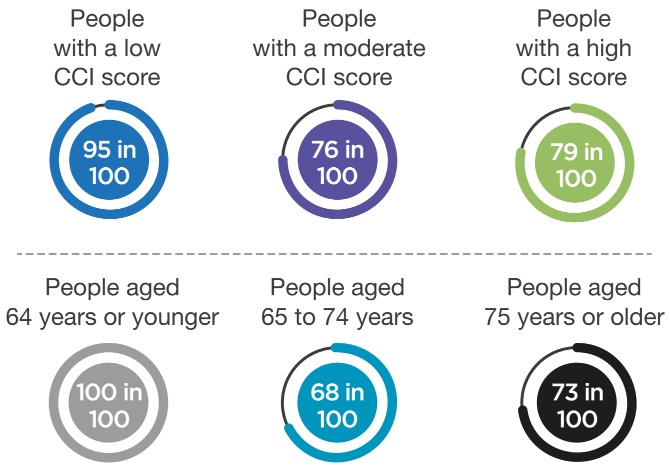

Safety

- Most people who took part in this study experienced side effects while taking bosutinib. Some of those side effects were considered to be 'severe'
  - A side effect is considered 'severe' when it limits daily activities, such as bathing and dressing, is disabling or is medically significant, or could be life-threatening, need hospital care, or cause lasting problems

Proportion of people who had severe side effects

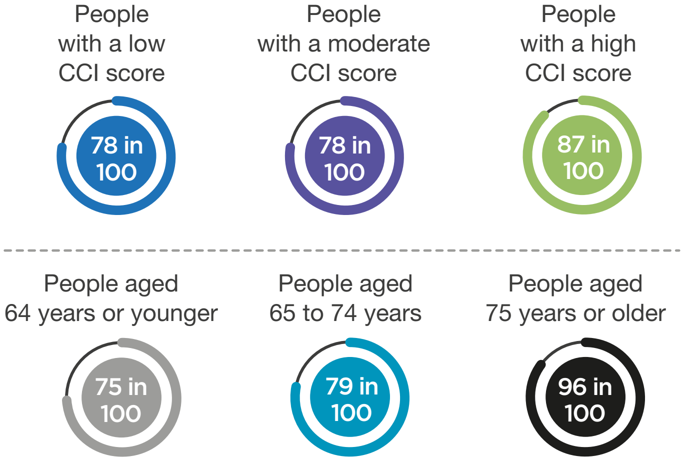

- To help reduce side effects, researchers sometimes recommended that people:
  - take less bosutinib, or
  - temporarily stop taking bosutinib

Additional information

More information can be found in the scientific article of this study, which you can access here:

View Scientific Article

For more information on clinical studies in general, please visit:

https://www.clinicaltrials.gov/ct2/about-studies/learn

https://www.cancerresearchuk.org/about-cancer/find-a-clinical-trial/what-clinical-trials-are

Bosutinib
<boh-SOO-tih-nib>

Chronic myeloid leukemia
<KRAH-nik MY-eh-loyd loo-KEE-mee-uh>

Dasatinib
<da-SA-tih-nib>

Imatinib
<ih-MA-tih-nib>

Nilotinib
<nye-LOH-tih-nib>

Tyrosine kinase inhibitor
<TY-ruh-seen KY-nays in-HIH-bih-ter>

Proportion of people who took a reduced dose of bosutinib due to side effects

People with a low CCI score	People with a moderate CCI score	People with a high CCI score
77 in 100	78 in 100	90 in 100

People aged 64 years or younger	People aged 65 to 74 years	People aged 75 years or older
80 in 100	70 in 100	89 in 100

Proportion of people who temporarily stopped taking bosutinib due to side effects

People with a low CCI score	People with a moderate CCI score	People with a high CCI score
71 in 100	81 in 100	90 in 100

People aged 64 years or younger	People aged 65 to 74 years	People aged 75 years or older
74 in 100	70 in 100	93 in 100

- Some people permanently stopped taking bosutinib due to side effects

Proportion of people who permanently stopped taking bosutinib due to side effects

People with a low CCI score	People with a moderate CCI score	People with a high CCI score
25 in 100	33 in 100	43 in 100

People aged 64 years or younger	People aged 65 to 74 years	People aged 75 years or older
22 in 100	39 in 100	46 in 100

Additional information

More information can be found in the scientific article of this study, which you can access here:

View Scientific Article

For more information on clinical studies in general, please visit:

https://www.clinicaltrials.gov/ct2/about-studies/learn

https://www.cancerresearchuk.org/about-cancer/find-a-clinical-trial/what-clinical-trials-are

Bosutinib
<boh-SOO-tih-nib>

Chronic myeloid leukemia
<KRAH-nik MY-eh-loyd loo-KEE-mee-uh>

Dasatinib
<da-SA-tih-nib>

Imatinib
<ih-MA-tih-nib>

Nilotinib
<nye-LOH-tih-nib>

Tyrosine kinase inhibitor
<TY-ruh-seen KY-nays in-HIH-bih-ter>

What were the main conclusions reported by the researchers?

- In this study, bosutinib worked for most people regardless of their age or if they had additional medical conditions
- These results also suggest that when taking bosutinib, older people or those with additional medical conditions may:
  - experience severe side effects more often, and
  - be more likely to stop taking bosutinib because of these side effects
- These results may help doctors and other health care professionals identify people with CML who may need to be monitored more closely when they take bosutinib

Who sponsored this study?

Pfizer Inc.

235 East 42nd Street NY, NY 10017

Phone (United States): +1 212-733-2323

Pfizer would like to thank all of the people who took part in this study.

Additional information

More information can be found in the scientific article of this study, which you can access here:

View Scientific Article

For more information on clinical studies in general, please visit:

https://www.clinicaltrials.gov/ct2/about-studies/learn

https://www.cancerresearchuk.org/about-cancer/find-a-clinical-trial/what-clinical-trials-are

Scientific Article

Rosti G, et al. Impact of Age and Comorbidities on the Efficacy and Tolerability of Bosutinib in Previously Treated Patients with Chronic Myeloid Leukemia: Results from the Phase 4 BYOND Study. Leukemia, 2023; doi: 10.1038/s41375-023-02080-y

Acknowledgements

Writing support for this summary was provided by Gemma Shay-Lowell, PhD, CMPP, Envision Pharma Group, Inc., and was funded by Pfizer.
